# New Orleans Exposition

**Published:** 1885-09-20

**Authors:** 


NEW ORLEANS EXPOSITION.—The Exposition opens in November next under a new organization called "The North, Central and South American Exposition." Improved facilities for getting to and from the grounds have been provided, and other improvements, suggested by the experience of the first exhibit, have been made. Everybody is rejoiced that the Exposition is to be again opened, and during the Winter months, as it was too great a show to be cut short in the brief time allowed to the first Exposition. The many who had not the opportunity to visit the first Exposition may now avail themselves of the opportunity to do so.

